# Automated, IMU-based spine angle estimation and IMU location identification for telerehabilitation

**DOI:** 10.1186/s12984-024-01366-1

**Published:** 2024-06-06

**Authors:** Huiming Pan, Hong Wang, Dongxuan Li, Kezhe Zhu, Yuxiang Gao, Ruiqing Yin, Peter B. Shull

**Affiliations:** 1 State Key Laboratory of Mechanical System and Vibration, Shanghai Jiao Tong University, Shanghai, 200240 China; 2 Tensor Health Inc, Shanghai, 200090 China

**Keywords:** Angle estimation, Classification, IMU, Telerehabilitation, Spine degeneration

## Abstract

**Background:**

Telerehabilitation is a promising avenue for improving patient outcomes and expanding accessibility. However, there is currently no spine-related assessment for telerehabilitation that covers multiple exercises.

**Methods:**

We propose a wearable system with two inertial measurement units (IMUs) to identify IMU locations and estimate spine angles for ten commonly prescribed spinal degeneration rehabilitation exercises (supine chin tuck head lift rotation, dead bug unilateral isometric hold, pilates saw, catcow full spine, wall angel, quadruped neck flexion/extension, adductor open book, side plank hip dip, bird dog hip spinal flexion, and windmill single leg). Twelve healthy subjects performed these spine-related exercises, and wearable IMU data were collected for spine angle estimation and IMU location identification.

**Results:**

Results demonstrated average mean absolute spinal angle estimation errors of 2.59^∘^ and average classification accuracy of 92.97%. The proposed system effectively identified IMU locations and assessed spine-related rehabilitation exercises while demonstrating robustness to individual differences and exercise variations.

**Conclusion:**

This inexpensive, convenient, and user-friendly approach to spine degeneration rehabilitation could potentially be implemented at home or provide remote assessment, offering a promising avenue to enhance patient outcomes and improve accessibility for spine-related rehabilitation.

*Trial registration:* No. E2021013P in Shanghai Jiao Tong University.

## Introduction

The widespread use of technology and the omnipresence of smartphones have contributed to a sedentary lifestyle, resulting in a growing prevalence of spine degeneration conditions among individuals who engage in prolonged periods of sitting with insufficient physical activity [1–4]. Spinal degenerative pathologies, including stenosis, spine curvatures, and cervical spondylosis, typically manifest with symptoms such as pain, neural disorders, and muscle rigidity [3]. Considering the risks associated with surgery and medication, the significance of rehabilitation exercise has gained prominence in recent years as a dominant treatment manner [5]. Nevertheless, the practicality of long-term hospital or rehabilitation center-based therapy has limitations, including cost and travel time, which can severely dampen patient motivation and potentially lead to therapy abandonment and inefficacy [6]. As a result, there has been a growing focus on developing telerehabilitation solutions.

Real-time assessment and feedback for telerehabilitation have emerged as crucial components in ensuring that patients can rapidly master the control of key muscle groups and determine the effectiveness of rehabilitation [7]. Two primary techniques, namely camera-based and wearable sensor-based solutions, have come to the forefront for assessing exercises and providing real-time feedback to patients. While camera-based approaches [8–10] offer the advantage of non-intrusive monitoring, they have limitations related to privacy, lighting, occlusion, computational resources, and cost, which restrict their practicality for real-time quantification of rehabilitation exercises. In contrast, wearable sensor-based solutions [6, 11–15], particularly those utilizing inertial measurement unit (IMU) sensors, have shown promise by offering low latency, robust accuracy, and minimal redundancy in capturing data about body segments.

IMUs have been used to estimate spinal rotation angles and joint kinematics for assessing axial spondyloarthritis during a set of five easy functional movements: hip flexion, back extension, lateral flexion, cervical rotation, and cervical flexion/extension [11]. Furthermore, the robustness of IMU-based assessments in evaluating spine control and functional movement quality across different days has been established through research involving the concurrent use of two IMUs during hip flexion/extension in the sagittal plane, shoulder rotation in the transverse plane, and combined movements [12, 13]. O’Grady et al. [14] demonstrated that IMU sensor-based systems reliably measure spinal mobility through a comprehensive study involving forty patients diagnosed with axial spondyloarthritis during spine flexion/extension, lateral flexion, and spine rotation. Additionally, IMUs were successfully validated for measuring spine angles in diverse daily-life scenarios, including standing, sitting, lifting, walking, and jogging, achieving an impressive regression performance [15]. While others have proposed using IMUs for estimating a limited set of spine movements, to date, there are no studies validating IMU spine movement estimation during rehabilitation exercises, including supine chin tuck head lift rotation, dead bug unilateral isometric hold, pilates saw, catcow full spine, wall angel, quadruped neck flexion/extension, adductor open book, side plank hip dip, bird dog hip spinal flexion, and windmill single leg [16–20].

However, within the context of telerehabilitation, the intricacies associated with system operation may pose challenges, potentially impacting patient motivation and self-efficacy [21, 22]. This challenge is particularly pronounced among elderly patients, who may face difficulty in wearing a full-body IMU set. A minimum of two IMUs is required for spine movement estimation, necessitating the patient to adjust IMU locations when switching exercises. The accurate detection of IMU locations plays a part in minimizing erroneous placement.

In this study, a 2-IMU system was proposed to identify the IMU locations and implement angle estimation for ten commonly prescribed spinal degeneration rehabilitation exercises. We hypothesized that angles estimated by the IMU-based method could reasonably assess the completeness and quality of rehabilitation exercises. This comprehensive approach seeks to advance the field of telerehabilitation, offering a promising avenue for improved patient outcomes and enhanced accessibility to professional-grade rehabilitation exercises.

## Methods

**Fig. 1:**
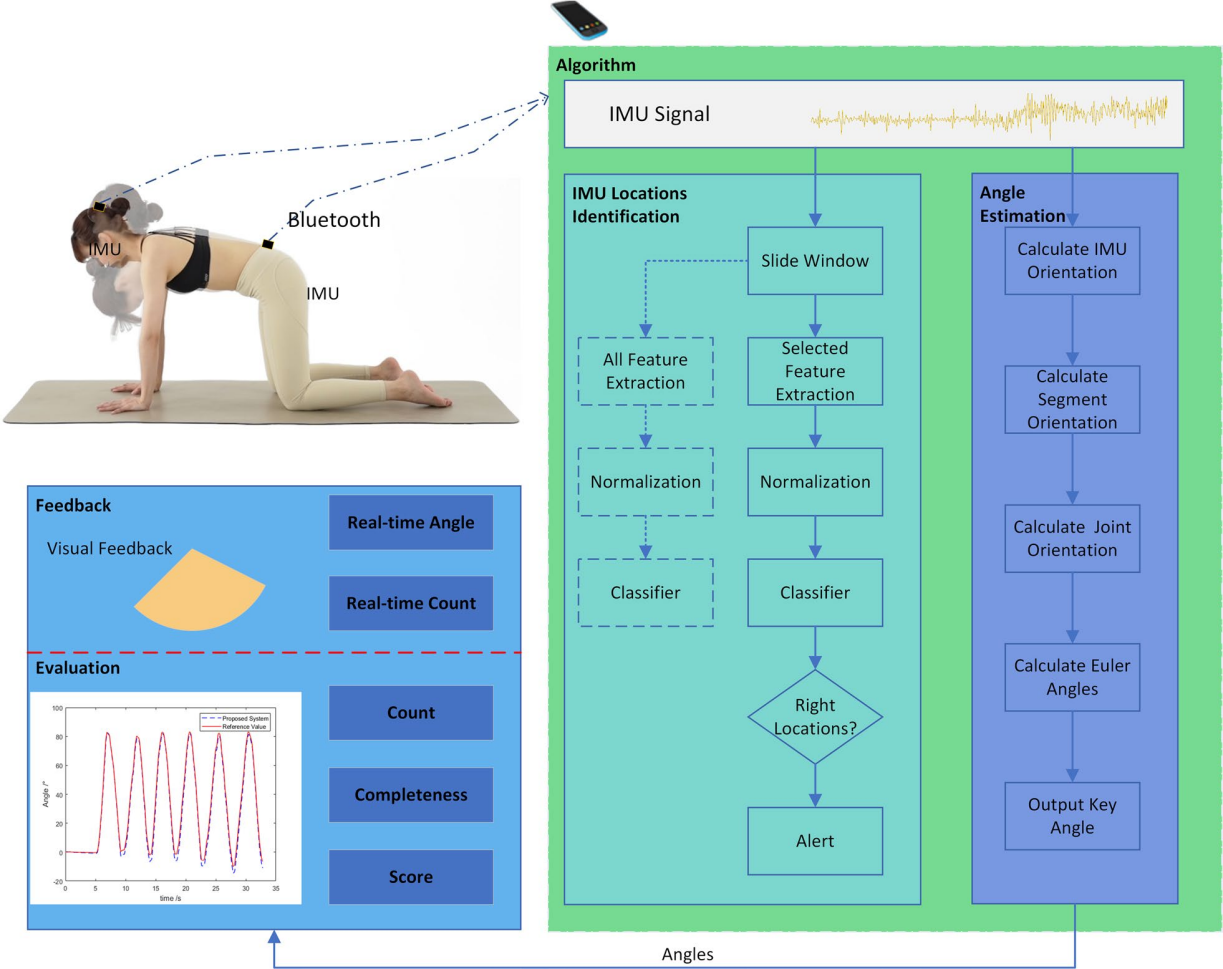
Proposed IMU-based telerehabilitation system. IMU sensors, worn by the user, connect to the cellphone via Bluetooth. The IMU signal serves for both IMU location identification and angle estimation. The estimated angle is then utilized for real-time feedback and offline evaluation. Detection of incorrect IMU placement prompts user alerts to ensure accurate monitoring

In this study, a 2-IMU system was designed to automatically identify the IMU locations and estimate crucial parameters for each exercise (Fig. 1). During rehabilitation exercises, IMU signals will be transmitted to a cellphone via Bluetooth. These signals will be utilized to identify the IMU locations and estimate the evaluation angle. The angles obtained serve the purposes of providing real-time visual feedback to guide the patient and counting repetitions, as well as facilitating offline evaluation.

### Exercises selection

**Fig. 2:**
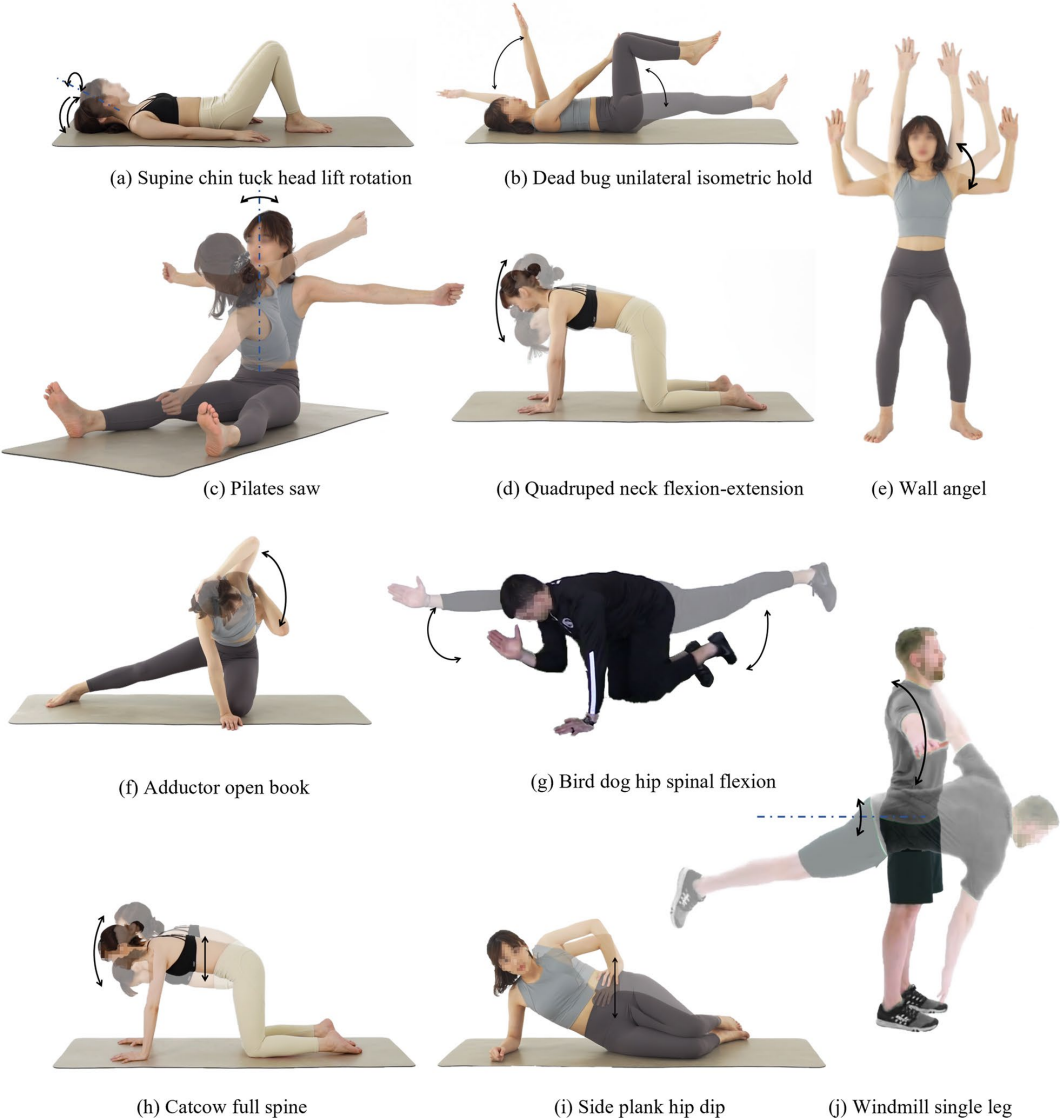
Selected rehabilitation exercises for evaluation. The motion range and primary movement for each exercise were illustrated. Arrows and dotted lines indicate the direction of motion and rotation axis, respectively. In supine chin tuck head lift rotation (SCTHLR), two movements, neck flexion/extension and head rotation, are performed sequentially. Other exercises feature parallel movements, such as shoulder flexion and hip flexion in the dead bug unilateral isometric hold, making transitions between solid and transparent poses. The repetitive nature of all movements constitutes a full cycle, encompassing the transition from a solid pose to a transparent pose and back to a solid pose

We carefully curated a set of ten commonly prescribed rehabilitation exercises for spinal degeneration, including supine chin tuck head lift rotation, dead bug unilateral isometric hold, pilates saw, catcow full spine, wall angel, quadruped neck flexion/extension, adductor open book, side plank hip dip, bird dog hip spinal flexion, and windmill single leg (Table 1 and Fig. 2) [16–20]. Each exercise specifically targets a distinct spine segment or the entire spine from a clinical rehabilitation perspective.

**Table 1:** Spine-related exercises for evaluation

Exercises	Spine segments	Kinematic angle	2 IMU locations
Supine chin tuck head lift rotation	CS	Neck rotation	Head, thorax
Dead bug unilateral isometric hold	LS	Hip flexion	Thigh, pelvis
Pilates saw	TS and LS	Thorax rotation	Thorax, pelvis
Catcow full spine	WS	Neck flexion	Head, pelvis
Wall angel	CS and TS	Shoulder abduction	Upper arm, thorax
Quadruped neck flexion/extension	CS	Neck flexion	Head, thorax
Adductor open book	CS and TS	Thorax rotation	Thorax, pelvis
Side plank hip dip	LS	Low back abduction	Thorax, pelvis
Bird dog hip spinal flexion	LS	Hip flexion	Thigh, pelvis
Windmill single leg	LS	Lumbar flexion	Thorax, pelvis

^1^Columns “Spine Segments”, “Kinematic Angle Estimation”, and “2 IMU locations” indicate the spine segments targeted by each exercise, the angles to be measured, and the locations of the 2 IMUs, respectively

^2^*CS* cervical spine, *TS* thoracic spine, *LS* lumbar spine, *WS* whole spine

From a kinematic standpoint, each exercise engaged one or more movements, with specific human segments identified as active components. These active segments, coupled with alternative movements, form the foundation for parameter measurement. For instance, in the supine chin tuck head lift rotation, the active segment is the head, and the two alternative movements are cervical spine rotation and flexion/extension.

It’s worth noting that the wall angel exercise engages the cervical and thoracic spine, while the active segment and movement are associated with upper arm and shoulder abduction/adductionâ€”segments not directly connected to the spine. However, owing to the close relationship between shoulder muscle groups and the cervical and thoracic spine, the wall angel exercise proves beneficial for enhancing upper spine mobility, potentially alleviating pain associated with spine degeneration [23].

### Movement selection

Before delving into the evaluation of rehabilitation exercises, careful consideration of the parameter(s) to be measured is paramount. Our choice of a two-IMU system strikes a balance between information richness and user-friendliness, allowing us to measure at least one movement without burdening users with the complexity of multiple sensors during telerehabilitation.

Certain rehabilitation exercises, such as pilates saw, wall angel, quadruped neck flexion/extension, and side plank hip dip, predominantly involve one movement each, while others encompass multiple movements (Fig. 2). Selecting the most relevant movement for assessing rehabilitation exercises is pivotal for minimizing the required IMUs.

Our movement selection adhered to the following principles:

- The motion range of the movement should be extensive enough to be measured by IMUs;
- The movement should be related to the target spine segment;
- The movement should be related to the active segment.

For example, in the case of supine chin tuck head lift rotation targeting the cervical spine, the alternative movements are cervical spine rotation and flexion/extension. Given the broader motion range of cervical spine rotation compared to flexion/extension, we opted to measure the neck rotation angle. IMUs strategically placed on the head and thorax were used to estimate neck rotation angle. Similar considerations guided the determination of the kinematic angle to be measured (Table 1).

### Data collection

#### Measurement system

Xsens MT system (MTw, Xsens Technologies, Netherlands) was used as the reference signals [24–26]. Custom-made IMU sensors (BNO055 IMU and STM32L475RCT6 MCU) were 9-axis IMUs, comprising a 3-axis accelerometer, a 3-axis gyroscope, and a 3-axis magnetometer. Both Xsens MT system and custom-made IMUs recorded data at 100Hz. The signal from the Xsens sensors was transmitted to a signal receiver and then sent to a computer for storage. Simultaneously, the custom-made IMU sensor was connected to a smartphone via Bluetooth, and the data was stored there.

Each sensor sends a 9-axis data frame with a sequentially increasing package number. The package numbers are used to synchronize between sensors and to check Bluetooth data loss. When two IMUs are connected to a smartphone using our custom application, the package numbers of both sensors are reset to zero. If the package number of one frame is not adjacent to the previous one, it indicates one or several lost frames, which are interpolated with the immediately preceding frame. The Received Signal Strength Indicator (RSSI) measures − 58.8 dBm when the sensor is placed 5 m away from the smartphone. The package loss rate at a distance of 3 m is 0.42%.

#### Experimental protocol

Twelve healthy subjects (mean age $23.4\pm 1.3$ years, height $176.7\pm 6.7$ cm, weight $65.4\pm 4.5$ kg, all males) participated in this study. All subjects provided informed consent before the experiment, and the research protocol adhered to the principles of the Helsinki Declaration. Approval for the experiment was obtained from the ethics committee of Shanghai Jiao Tong University (No. E2021013P). In this study, subjects were recruited to gather data for system development.

Before wearing the IMU sets, all sensors were placed on a table for 10 seconds to record gyroscope bias, a parameter later compensated during subsequent analysis.

Each IMU was secured by a research facilitator with Velcro straps. Before initiating motion in each trial, subjects held the initial pose statically for 3 s to establish IMU-to-segment alignment (Equation 1). Subject performed five repetitions of each of the 10 exercises. Adequate breaks were provided during trials to prevent muscle fatigue.

### IMU locations identification

The automated identification of IMU locations reduces the likelihood of improper IMU placement, particularly during transitions between exercises. Patients with specific spinal segment degeneration may be prescribed multiple rehabilitation exercises, necessitating adjustments to IMU locations when transitioning from one exercise to another (see Table 1). IMU location detection serves to remind users when the placement does not align with the current exercise being performed.

The process of identifying IMU locations involves several steps: sliding window analysis, feature extraction, feature selection, normalization, and classification (Figs. 1 and 4).

#### Classification model

We employed Linear Discriminant Analysis (LDA) as the supervised learning algorithm for IMU location identification. LDA functions as a linear classifier, aiming to maximize the separation between the means of distinct classes while minimizing the variance within each class. Its linear nature demands relatively modest computational resources [27, 28] and facilitates seamless implementation and deployment on smartphones [29, 30]. The utilization of LDA ensures precision and enhances the efficiency of IMU location identification, making it a suitable choice for our dynamic real-world rehabilitation scenarios. Consequently, we exclusively conducted experiments using LDA.

#### Feature extraction and normalization

Since IMU location identification was intended to occur at the initial stage of real-world rehabilitation exercises, only the data from the first ten seconds (including the calibration process) of each experimental trial were utilized for model training. This approach also ensures a balance of samples for various exercises.

The IMU data underwent segmentation into 3-s windows, each consisting of 300 data points, with a 0.01-s stride (equivalent to 1 data point) (see Fig. 4). For the initial 10 seconds of a trial, we obtained $(10-3)/0.01+1 = 701$ window. Therefore, a total of $701\times 12\times 10\times 5=420600$ windows could be obtained for all twelve subjects, ten exercises, and five IMU locations. This segmentation allows for efficient processing without being excessively long or too short for adequate information for IMU location identification [31].

Following segmentation, both time-domain and frequency-domain features were extracted from each window. Time-domain features included mean, standard deviation, root mean square, median, skewness, kurtosis, waveform length, zero crossings, slope sign changes, mean absolute value, interquartile range, and median absolute deviation. Additionally, the frequency-domain featureâ€”spectral peak frequency and spectral peak indexâ€”contributed, resulting in a total of 14 features for one IMU channel within a window. Consequently, the feature count was $14\times 6=84$ for one IMU window (3-axis accelerometer and 3-axis gyroscope). The labels, representing the five IMU locations, were numerically encoded from 1 to 5 for classification purposes.

The extracted features were subsequently normalized to follow a standard normal distribution, with a mean of 0 and a standard deviation of 1, facilitating faster training and convergence.

#### Feature selection

Initially, all features were included in the training and testing of the classification model. However, recognizing the imperative for real-time efficiency, we refine the feature channels to reduce the time for feature extraction and classification. Features with marginal impact on classification underwent systematic pruning, facilitating a streamlined and low-delay classification process.

In pursuit of this efficiency, an eXtreme Gradient Boosting (XGBoost) classifier was employed to select dominant feature channels. Through its boosting algorithm, XGBoost iteratively constructed a series of weak learners, each rectifying its predecessor’s errors. Additionally, XGBoost’s capability to assign importance scores to each feature channel helps to select feature channels. This feature ranking ability enabled an assessment of feature relevance, facilitating the selection of the most influential channels for our classification task. Leveraging this property, less crucial channels were pruned, while those with the highest importance were retained, resulting in a refined set of feature channels tuned for IMU location identification. Although reducing the number of features may entail the loss of some useful data information for classification, it represents a balance between precision and real-time efficiency. In this study, we opted to select the most crucial features, whose cumulative importance contributed to $90\%$ of the total importance, for classification.

#### Training and test strategy

To rigorously assess the performance of the classification model, we employed the 80/20 split strategy. Data from nine subjects were selected as training data, while data from the remaining three subjects were allocated as test data. To mitigate accidental biases, we randomly divided the subjects into training and test sets ten times.

#### Evaluation

The classification accuracy was calculated for each IMU location, defined as the ratio of correctly classified windows to the total number of windows. The overall classification accuracy was determined by computing the mean value across all ten iterations and ten exercises. Additionally, confusion matrices were generated to delve deeper into the model’s effectiveness at each IMU location.

### Angle calculation

Compensating for the gyroscope bias is a prerequisite before utilizing the original IMU data, and we achieved this through static calibration to calculate the gyroscope bias.

**Fig. 3:**
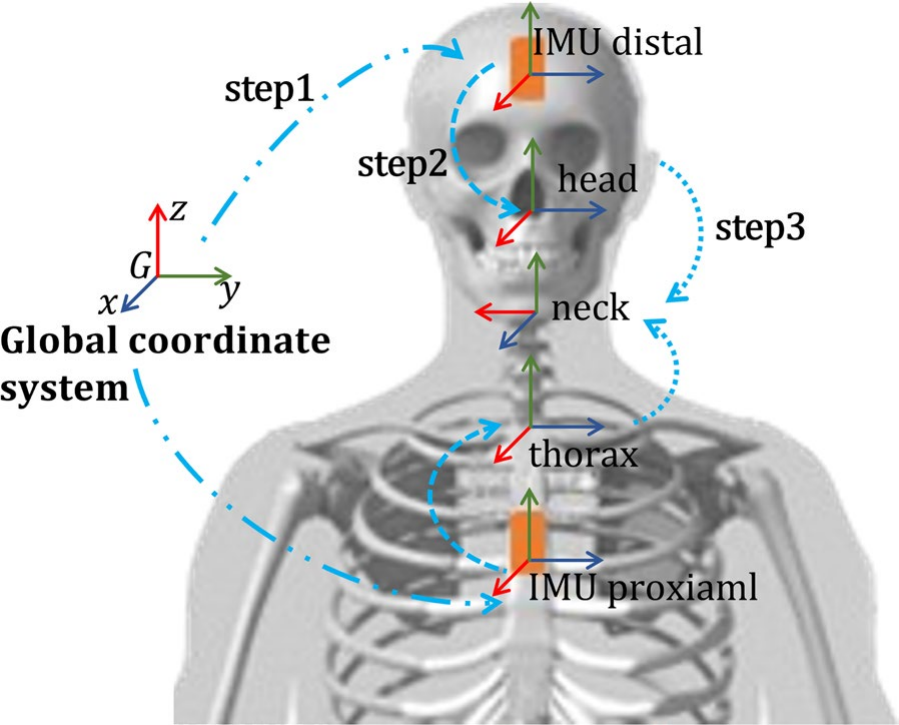
The procedure for joint angle estimation involves a four-step algorithm. Firstly, the orientation of the sensors relative to the global coordinate system is estimated. Secondly, calibration is performed to determine the relative orientation of human body segments in relation to their corresponding IMUs. Thirdly, the relative orientation of the distal segment with respect to the proximal segment is calculated. Finally, the joint angle is derived from the relative orientation. It’s important to note that the fourth step is not illustrated in the figure

The angle calculation procedure unfolds in four steps (Fig. 3) [32–34].

First, we computed the orientation quaternions of the proximal and distal IMU local coordinate systems at each time frame ($\ ^G\pmb q_{\mathrm {IMU\_Proximal}}$ and $\ ^G\pmb q_{\mathrm {IMU\_Distal}}$) using the accelerometer, gyroscope, and magnetometer data. To achieve this, we employed the direct complementary filter, a nonlinear observer on *SO*(3), to effectively fuse the IMU data [35, 36].

Second, the orientation of proximal and distal segments with respect to the global coordinate system was calculated through the equation:

1 $$\begin{aligned} \ ^G\pmb q_{\textrm{Segment}}=\ ^G\pmb q_{\textrm{IMU}} \otimes \ ^{IMU}\pmb q_{\textrm{Segment}} \end{aligned}$$

where $\ ^G\pmb q_{\textrm{Segment}}$ is the orientation quaternion of the segment with respect to the global coordinate system (G), $\ ^{G}\pmb q_{\textrm{IMU}}$ is the orientation quaternion of the adhering IMU with respect to the global coordinate system calculated in the previous step, $\ ^{IMU}\pmb q_{\textrm{Segment}}$ is the IMU-to-segment alignment transformation, assumed to be invariant during an exercise trial. Various methods, such as static calibration [37] and functional calibration [38], can be employed to calculate $\ ^{IMU}\pmb q_{\textrm{Segment}}$. In our study, the initial pose of each exercise was maintained as static for 3 s, during which the data was used to calculate the IMU-to-segment alignment transformation. This static calibration simplified the procedure as it did not require the subject to maintain specific poses like N-pose[37] or T-pose [39]. The calibration method used didn’t necessitate the user to hold a distinct pose and then transition to the initial pose. Additionally, this method maintains the initial pose as the zero-point, providing an intuitive reference.

Third, the joint quaternion $\pmb q_{\textrm{Joint}}$ was calculated from the relative orientation of adjacent segments:

2 $$\begin{aligned} \pmb q_{\textrm{Joint}} =(\ ^G \pmb q_{\textrm{Segment Proximal}})^*\otimes \ ^G \pmb q_{\textrm{Segment Distal}} \end{aligned}$$

Here, $\ ^G \pmb q_{\textrm{Segment Proximal}}, \ ^G \pmb q_{\textrm{Segment Distal}}$ represent the orientation of proximal and distal segments in the global coordinate system (calculated from Eq. (1)).

Finally, we computed the corresponding rotation matrix $\pmb R_{\textrm{Joint}}$ from the joint orientation quaternion $\pmb q_{\textrm{Joint}}$, and the key parameter of each exercise (i.e., the selected kinematic angle to be measured) was obtained from the rotation matrix $\pmb R_{\textrm{Joint}}$.

The key parameter of each exercise, representing the selected movement to be measured, is derived from the rotation matrix $\ ^G\pmb R_{Joint}$ by extracting Euler angles using the *ZXY* sequence:

3 $$\begin{aligned} \alpha =\arctan \frac{\pmb R_{13}}{\pmb R_{33}}, \beta =\arcsin {(-\pmb R_{23})}, \gamma = \arctan {\frac{\pmb R_{21}}{\pmb R{22}}} \end{aligned}$$

Here, $\alpha$, $\beta$, and $\gamma$ represent the y-axis yaw, x-axis pitch, and z-axis roll angles of the joint connecting the distal and proximal segments. Following the coordinate definition conventions in [40, 41], flexion, rotation, and abduction correspond to $\gamma$, $\alpha$, and $\beta$, respectively.

### Deployment

The angle estimation algorithm underwent initial offline implementation using MATLAB to validate its feasibility. Subsequently, it was adapted for real-time implementation to offer instantaneous measurement for telerehabilitation. The offline implementation was conducted on MATLAB 2020a, while the real-time implementation was performed on Redmi Note 12 (with Android 12.0).

**Fig. 4:**
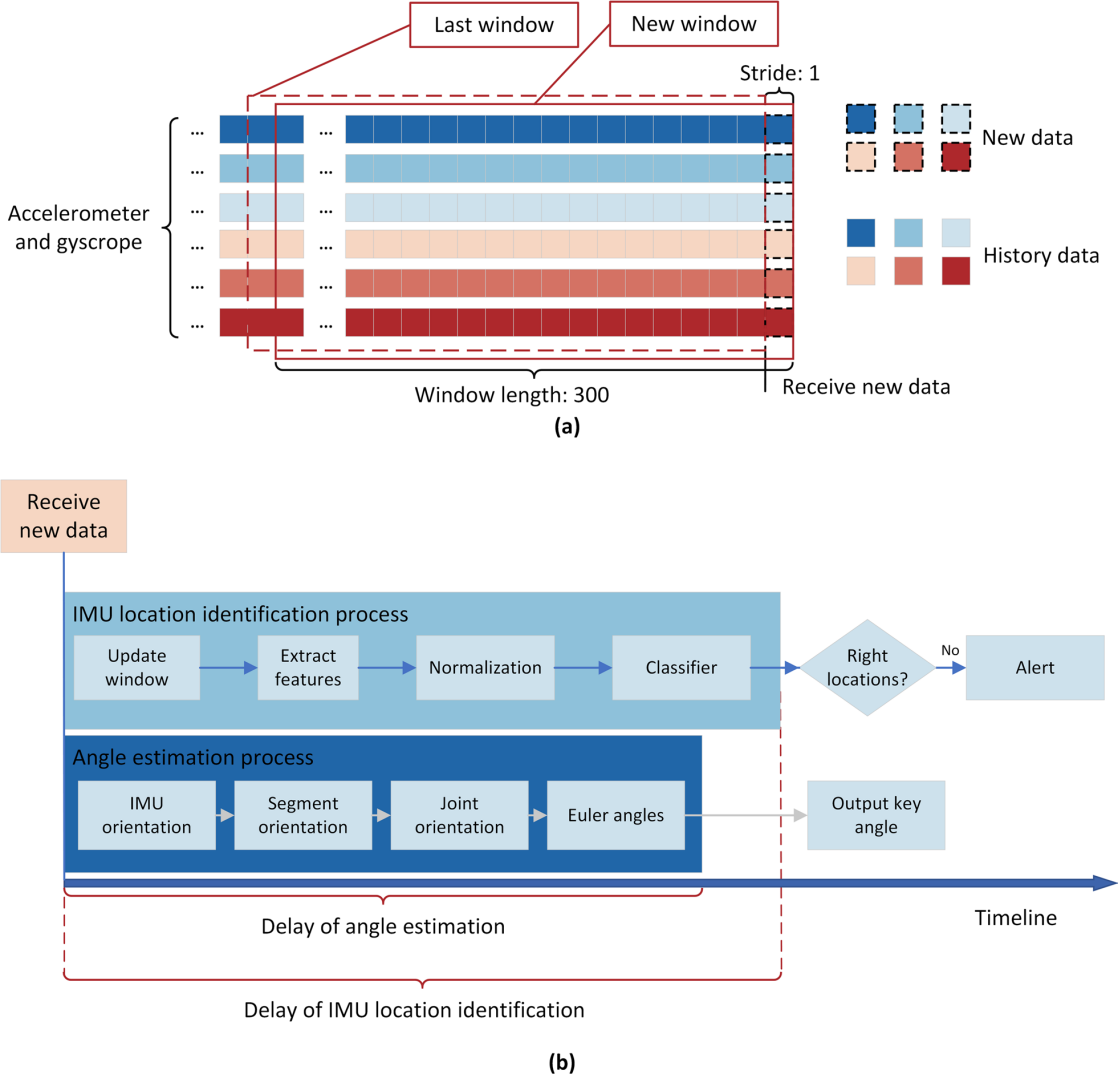
**a** Slide window on accelerometer and gyroscope of one IMU. **b** Delay definitions of angle estimation and IMU location identification

The delay in our real-time implementation of angle estimation, measured from the collection of IMU data to the calculation of the angle (see Fig. 4), was tested and found to be $48\textrm{ms}$. This delay is even shorter than the duration of visual staying (0.1$\sim$0.4s), ensuring the practicality and effectiveness of the system in telerehabilitation scenarios. The delay for the IMU location identification, measured from the collection of IMU data to the output of identified IMU locations (see Fig. 4), was tested and found to be $97\textrm{ms}$ when 36 features were selected.

Following offline training and evaluation, the LDA model for the selected features and the corresponding feature scaler were deployed on smartphones. To simplify the implementation, the parameters of the LDA model (coefficient matrix and intercept vector) and the scaler (mean vector and deviation vector) were exported and saved in the JavaScript project responsible for generating the cellphone app. The predicted category *y* of an extracted feature $\pmb {x}$ is:

4 $$\begin{aligned} \begin{aligned} \pmb {p}&= \textrm{Softmax}\left( \textbf{W}\cdot \frac{\pmb x-\pmb \mu }{\pmb \sigma }+\pmb b\right) \\ y&= \textrm{argmax}(\pmb p) \end{aligned} \end{aligned}$$

Here, $\pmb {x},\pmb {\mu },\pmb {\sigma }\in \mathbb {R}^d$ represent the extracted feature vector, mean values of the scaler, and standard variance of the scaler, respectively, where *d* is the dimension value (36 in this study). $\pmb {W}\in \mathbb {R}^{l\times d},\pmb {b}\in \mathbb {R}^{l}$ denote the weights and intercepts of the LDA, where *l* is the number of output categories (5 in this study). $p\in \mathbb {R}^{l}$ is a vector where *i*-th element represents the probability that $\pmb {x}$ belongs to category *i*.

## Results

### Classification results

**Fig. 5:**
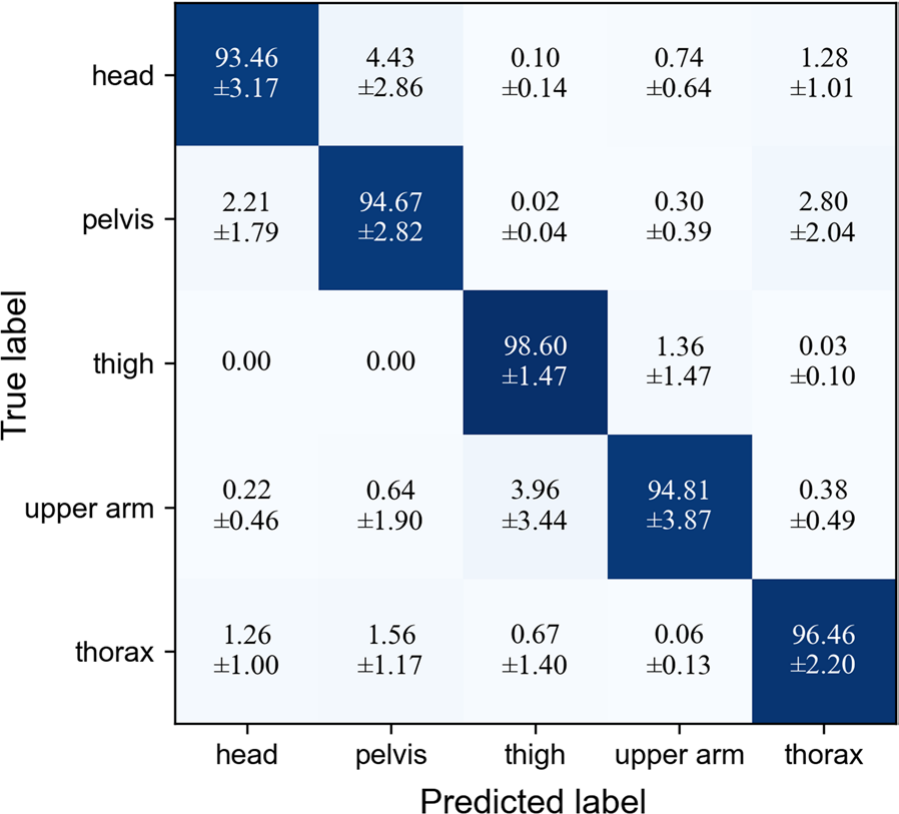
Normalized confusion matrix illustrating the average classification accuracy and standard deviation over ten randomly split datasets, encompassing all ten exercises. The mean classification accuracy was calculated to be 95.60%

To evaluate the LDA model, we employed an 80/20 split strategy. In each training and evaluation cycle, data from nine subjects were utilized as training data, with the remaining data from three subjects serving as testing data. The average results across ten exercises and ten random splits were depicted in the form of a confusion matrix (Fig. 5). The overall average classification accuracy was found to be 95.60%. These results demonstrate a significant level of accuracy.

**Fig. 6:**
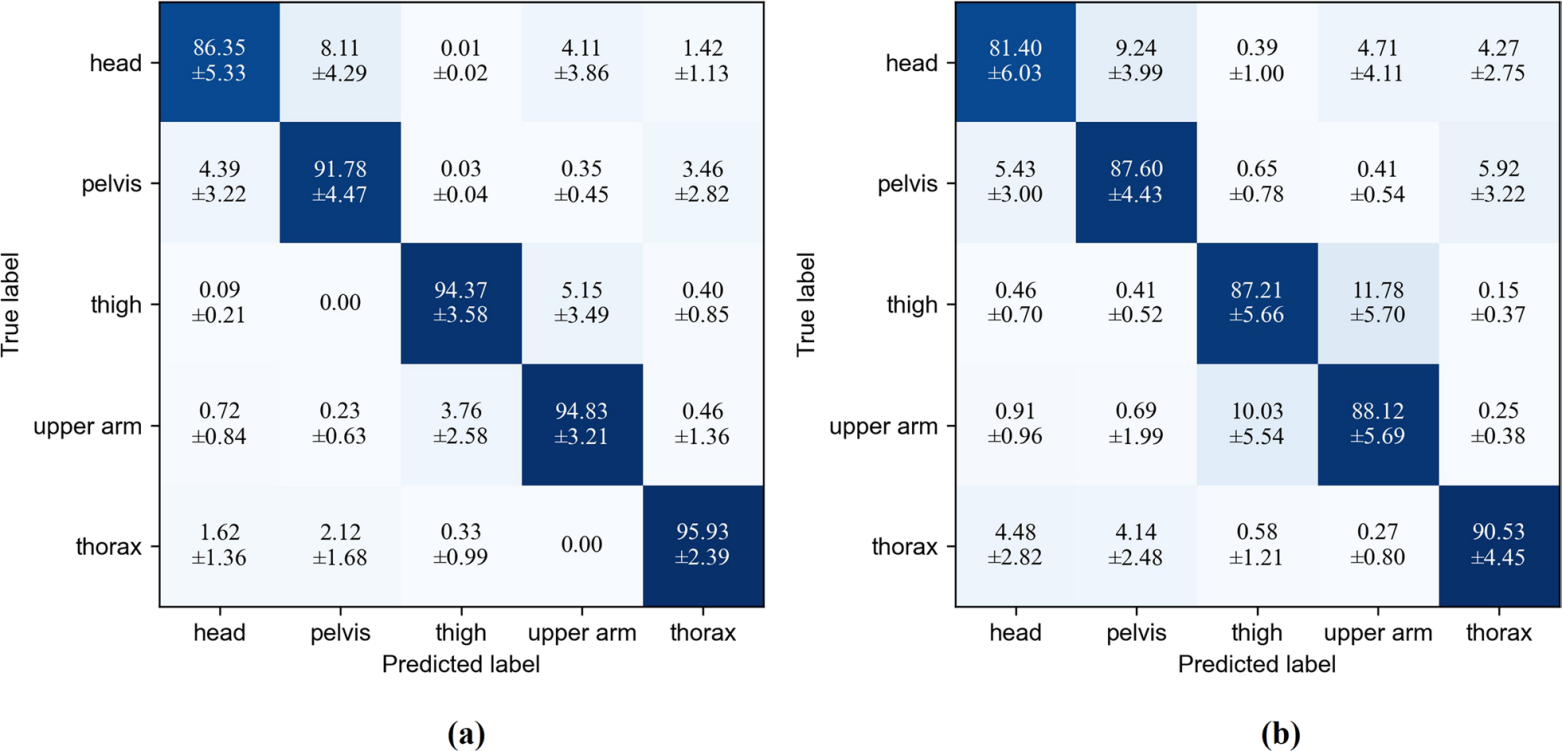
Normalized confusion matrix illustrating the average classification accuracy based on the selection of 36 features. **a** Results from the 36 most significant feature channels identified by XGBoost. **b** Results from a randomly selected subset

Given the computational load associated with utilizing all 84 feature channels for real-time classification, we opted for XGBoost to identify the most influential channels. To account for variance in feature importance when datasets change, we employed the entire dataset for the XGBoost feature selection process. Following the ranking of feature channels based on importance, we selected the top 36 channels, whose cumulative importance amounted to 90%, for retraining and testing the LDA model. The effectiveness of XGBoost-selected features was compared with 36 randomly selected features (see Fig. 6). XGBoost feature selection demonstrates competence in identifying dominant feature channels.

**Fig. 7:**
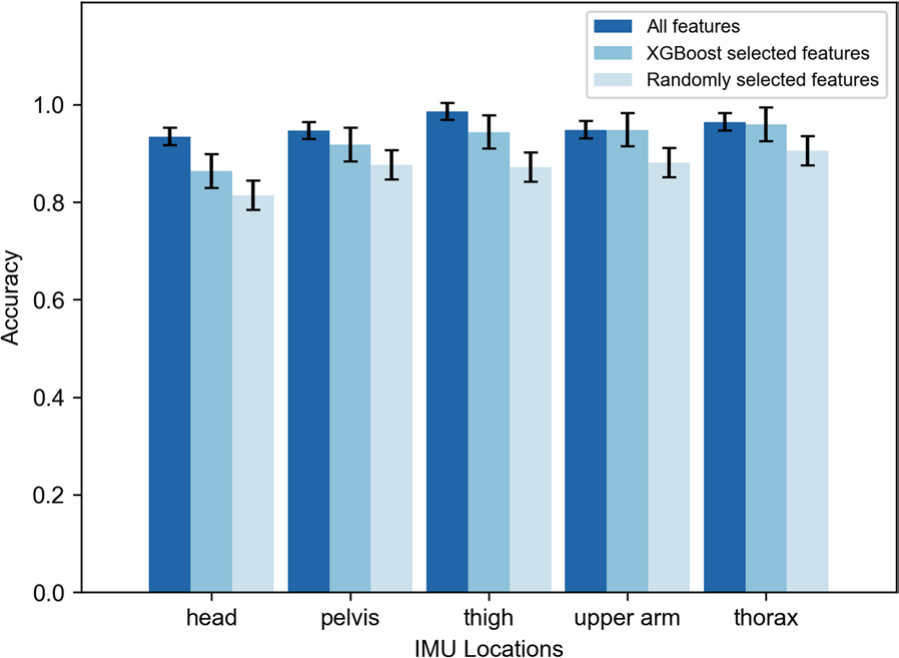
Comparison of classification accuracy among different feature sets. All features, features selected by XGBoost, and features randomly selected were used to train the LDA model separately. XGBoost reduced the feature channels from 84 to 36, with an average accuracy decrease from 95.60 to 92.97%. The accuracy for randomly selected 36 features was 86.03%

A marginal decrease in classification accuracy was observed, with the overall mean accuracy dropping from 95.60 to 92.97% (Fig. 7). Slight decreases were noted across all IMU locations. The results of the One-way Analysis of Variance (ANOVA) indicated that no significant difference was observed in the upper arm and thorax IMUs ($p>0.05$).

**Fig. 8:**
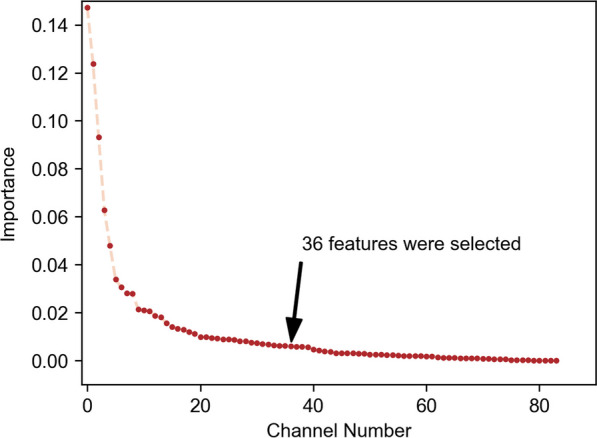
The sorted importance of feature channels. The thirty-six most important feature channels were selected, whose accumulative importance was up to 90%

The scree plot illustrating the importance of features was observed (see Fig. 8). The selected features comprised mean absolute value (6 channels), median (6 channels), mean (5 channels), index of spectral peak (4 channels), spectral peak frequency (3 channels), root mean square (3 channels), waveform length (3 channels), slope sign changes (2 channels), interquartile range (2 channels), median absolute deviation (1 channel), and standard deviation (1 channel) (see Appendix).

Incorporating the entire process, encompassing feature extraction and model prediction, the computational time for selected features decreased from 212 to $97\textrm{ms}$ (a $54.2\%$ reduction) on our utilized smartphone. This enhancement in computational efficiency notably mitigated delays, ensuring smoother system operation. Such strategic feature selection not only improves the efficiency of real-time exercise classification but also preserves overall accuracy.

### Angle estimation results

**Fig. 9:**
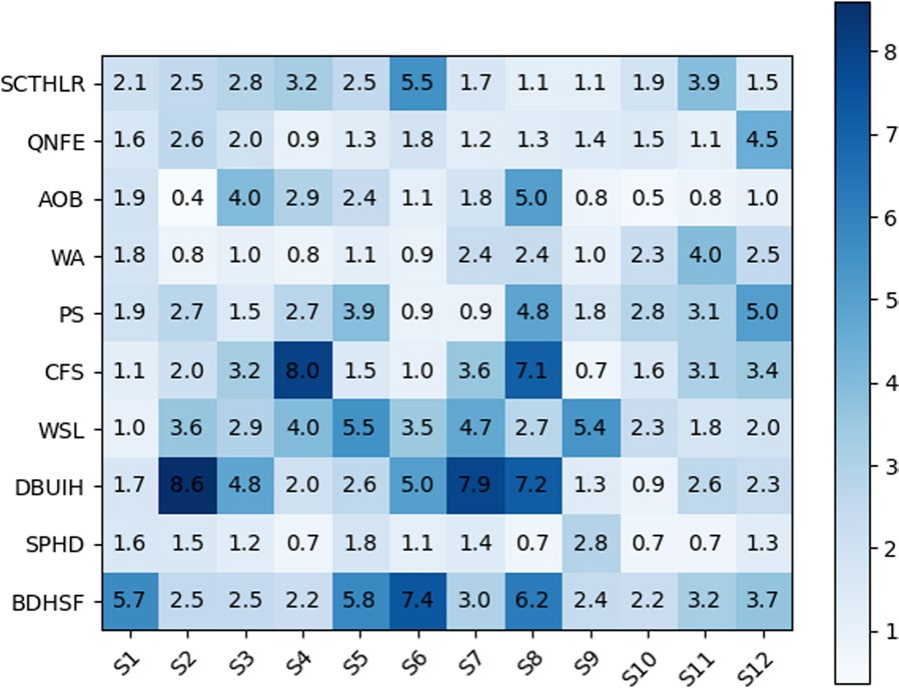
Mean absolute error (MAE) of angle estimation across all subjects and all exercises. The maximum and minimum MAE values were $8.60^\circ$ and $0.36^\circ$ respectively

**Fig. 10:**
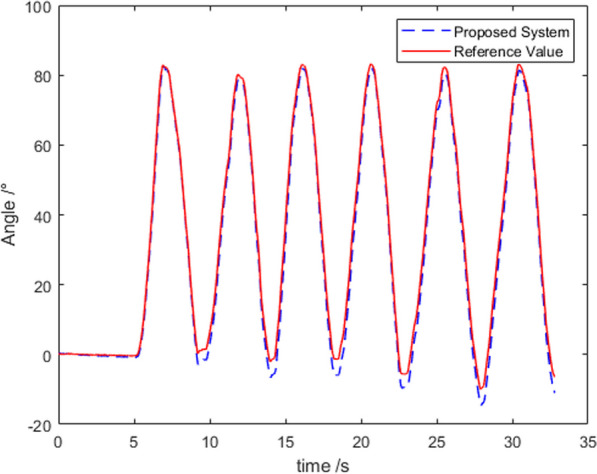
Representative trial showing the angle estimation of supine chin tuck head lift rotation. The mean absolute error (MAE) was $2.43^{\circ }$

The angle estimation results obtained from the IMU-based method were compared against the reference. Mean absolute error (MAE) was used as the metric for the angle estimation of our proposed system (Fig. 9). An example of the angle estimation results (Fig. 10) depicts the wall angel during a five-loop trial. The initial smooth period represents the static pose for about 3 s, which is crucial for obtaining the initial pose. In this trial, the mean absolute error (MAE) for the IMU-based angle estimation is $2.43^{\circ }$. This metric quantifies the disparity in the angles estimated by the custom-made IMUs and the reference.

The collective MAE, peak error, and mean velocity error across all trials were $2.59\pm 0.93^{\circ }$, $0.97\pm 5.62^{\circ }$ and $6.90\pm 4.75^\circ /\textrm{s}$, showcasing overall accuracy. The maximum and minimum MAE values were $8.60^\circ$ and $0.36^\circ$ respectively. These results underscore the high precision of the IMU-based spine angle estimation method, effectively validating its real-time applicability during rehabilitation exercises.

#### Angle estimation accuracy for each exercise

**Fig. 11:**
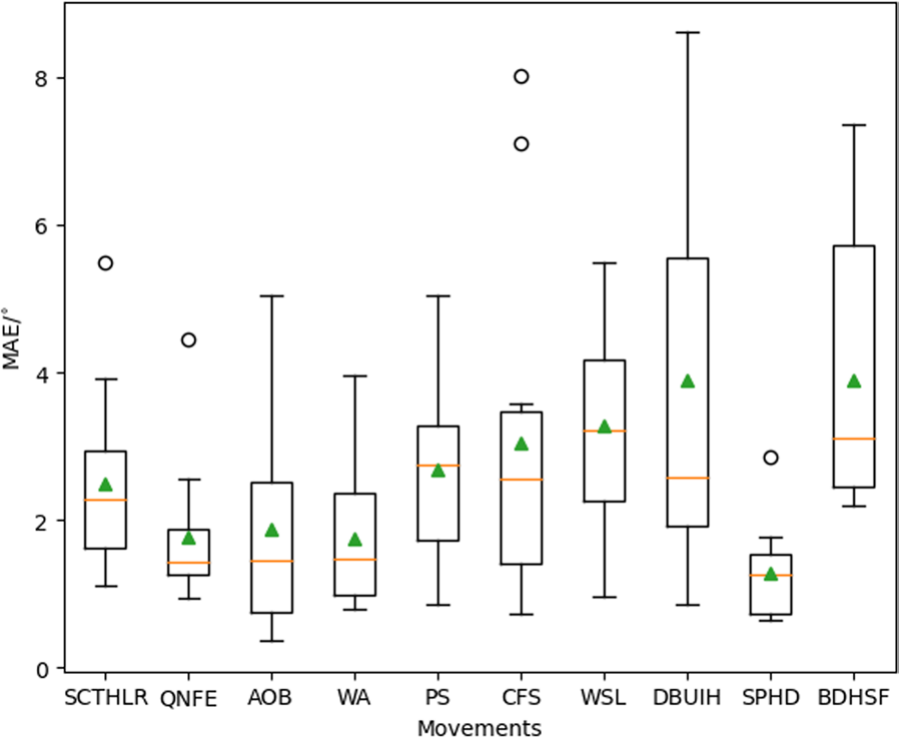
MAE of angle estimation for each of the 10 spine-related exercises. The overall average MAE across all ten exercises was $2.59\pm 0.88^{\circ }$

The accuracy of the IMU-based angle estimation method was systematically assessed for each specific exercise. Across twelve subjects, the MAEs for each of the ten exercises were calculated (Fig. 11). Across all ten exercises, the mean and median MAE values were $2.59\pm 0.88^{\circ }$ and $2.2\pm 0.71 ^{\circ }$ respectively, with the maximum value of and $3.90^\circ$.

ANOVA indicated a non-significant difference in the angles estimated by the IMU-based method across the diverse set of rehabilitation exercises ($p>0.05$). This finding underscores the inter-exercise reliability of our approach, emphasizing its consistent accuracy across a spectrum of rehabilitation exercises.

#### Angle estimation accuracy for each subject

**Fig. 12:**
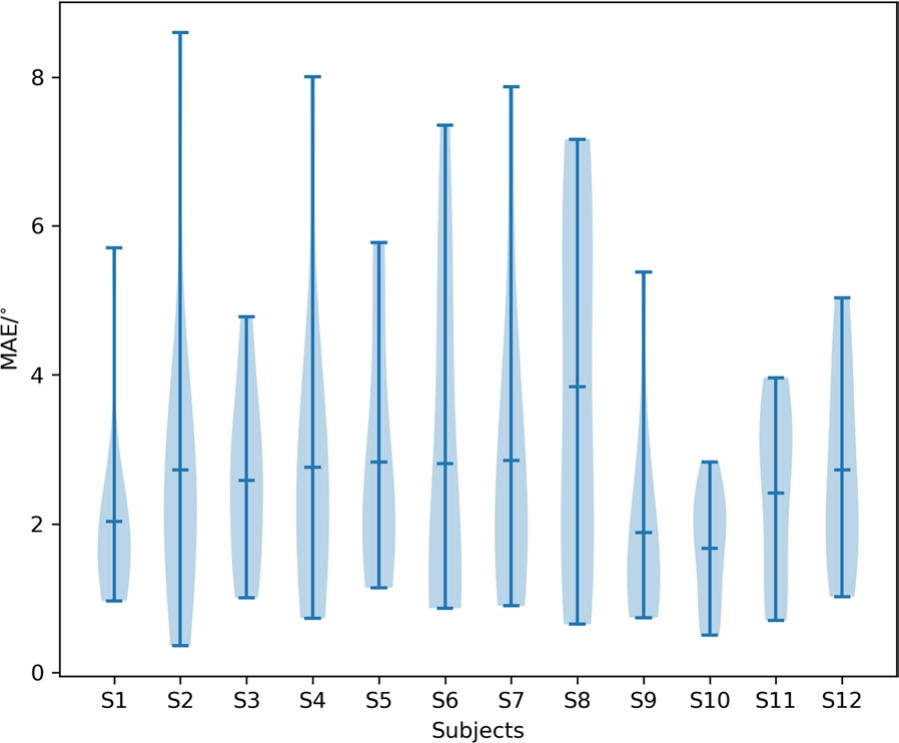
MAE of angle estimation for each of the 12 subjects. The overall average MAE across all 12 subjects was $2.59\pm 0.54^{\circ }$

The accuracy of the IMU-based angle estimation method was also individually assessed for each subject to investigate its precision across diverse individuals. The MAEs across ten exercises for each subject were calculated (Fig. 12). Across twelve subjects, the mean and median MAE values were $2.2\pm 0.64 ^{\circ }$ and $2.59\pm 0.54^{\circ }$ respectively, with maximum values of $3.77^{\circ }$.

ANOVA revealed a non-significant difference in the angles estimated by the IMU-based method across subjects ($p> 0.05$). This highlights our approach’s robustness and inter-subject reliability, emphasizing its consistent accuracy across a diverse population.

## Discussion

This study introduces an IMU-based telerehabilitation system designed for spine degeneration. Initially, ten commonly prescribed exercises targeting spine-related rehabilitation were chosen, and the specific angle to be measured for each movement was identified. The identification of IMU locations was accomplished through the implementation of a LDA model. Additionally, feature selection, employing XGBoost, was applied to diminish the number of features, thereby reducing computational demands.

The performance of our IMUs closely matched the reference system [24–26], showing that the system was capable of being used for spine angle estimation. The consistently high accuracy observed in angle estimation across various professional exercises and subjects indicates the reliability and robustness of the angle estimation algorithm. The average MAE across all subjects was $2.59^\circ$ and the range of errors was $0.42^\circ$ to $8.61^\circ$, suggesting that the IMU system was capable of providing reasonable measurements. It is a promising tool for monitoring and guiding rehabilitation exercises. The delay of $48\textrm{ms}$ in our real-time implementation of angle estimation ensures timely feedback, meeting the criteria for practical use. In sum, the IMU-based method emerges as a valuable asset for advancing rehabilitation technology.

The versatility of our system is further underscored in the evaluation of IMU identification classification. The LDA model exhibits satisfactory accuracy in distinguishing between different IMU locations. In our strategic feature selection process, we reduced the original 84 channels to the top 36, accounting for 90% cumulative importance. This reduction had little impact on the classification accuracy of most exercises. This computational efficiency is crucial for real-world deployment, ensuring the system’s practicality.

**Table 2:** Comparison of IMU-based spine angle estimation studies

Study	Real-time?	How many exercises?	Rehabilitation exercises?
Franco1 et al. [11]	No	3	Functional movements
Graham et al. [12]	No	3	Functional movements
Beange et al. [13]	No	1	Functional movement
Oâ€™Grady et al. [14]	No	3	Functional movements
Petropoulos et al. [42]	Yes	1	Functional movement
Darragh et al. [43]	No	1	Rehabilitation movement
Proposed study	Yes	10	Rehabilitation movements (Fig. 2)

Instead of using a full-body motion capture system [44–47], we opted for a two-IMU system to balance information richness and user-friendly application. We have endeavored to ensure right placement of the IMUs through automated identification of their locations. Although this system holds promise for application in patient settings, further research is warranted.

In comparison to prior studies using IMU-based methods for spine angle estimation [11–14, 42, 43, 48], our evaluation extends to a broader range of rehabilitation exercises, including a set of commonly clinically prescribed exercises for cervical, thoracic, and lumbar spine rehabilitation (Table 2). The real-time implementation of the angle estimation algorithm and integration of a machine learning model for IMU location identification further enhance the system’s applicability in real-life scenarios.

Despite achieving high overall accuracy in angle estimation, it is crucial to acknowledge the observed variability in accuracy in several trials. Several factors may contribute to this variability: (1) changes in the alignment transformation between IMU and segment during the exercise, (2) the complexity of the exercise, (3) variability in individual performance, and (4) the potential impact of sensor placement. Understanding these factors is vital for refining the system and addressing challenges associated with real-world applications. Additionally, the observed variability in the classification accuracy of individual exercises suggests that the complexity of the exercise and individual performance variations contribute to classification challenges. Further exploration and strategies to mitigate these variations will be instrumental in enhancing the robustness and reliability of the classification model.

It is essential to acknowledge the limitations of the study. The evaluation was conducted with a relatively small sample size, and the inclusion of a more diverse population could provide additional insights into the system’s performance. Additionally, the study focused on specific rehabilitation exercises, and the system’s generalizability to a broader range of exercises warrants exploration. Also, the system’s performance in real-life scenarios, such as telerehabilitation for patients, remains to be evaluated. The system was built and evaluated based on healthy individuals. The performance is likely to diminish for patients owing to the distinction between patients and healthy people. Transfer to patients requires corresponding adjustments for training the classification model to suit their characteristics.

Several participant factors could influence system usability, ease of sensor placement, and ease of software use including age, experience with the technology, and familiarity with the sensors which were beyond the scope of this study but should be carefully studied in future work.

## Conclusion

In conclusion, this study successfully validated the real-time IMU-based spine angle estimation approach during rehabilitation exercises. The proposed 2-IMU system effectively estimated critical parameters for a diverse range of rehabilitation exercises and provided real-time IMU location identification. The angle estimation algorithm showcased low delay and high accuracy across various exercises and subjects, positioning it as a promising tool for monitoring and guiding rehabilitation protocols. The integration of a machine learning model for IMU location identification further enhances the system’s adaptability to real-life scenarios.

Moving forward, the system holds potential for refinement and expansion to encompass a broader spectrum of rehabilitation exercises, catering to the diverse needs of patient populations. Collaborative endeavors with healthcare professionals and integration into telerehabilitation platforms could facilitate its seamless adoption in clinical settings. The proposed system could be a valuable asset in advancing rehabilitation technology.

## Data Availability

Data sets generated during the current study are available from the corresponding author on reasonable request.

## Appendix A: feature channels selected by XGBoost

**Table:**

Importance rank	Sensor	Axis	Feature
1	Gyroscope	y	Spectral peak frequency
2	Gyroscope	y	Mean absolute value
3	Gyroscope	x	Median
4	Gyroscope	y	Root mean square
5	Gyroscope	x	Mean absolute value
6	Gyroscope	z	Interquartile range
7	Gccelerometer	y	Spectral peak frequency
8	Accelerometer	z	Waveform length
9	Gyroscope	y	Spectral peak index
10	Gyroscope	z	Median absolute deviation
11	Accelerometer	z	Spectral peak frequency
12	Accelerometer	y	Spectral peak index
13	Accelerometer	y	Root mean square
14	Gyroscope	z	Root mean square
15	Accelerometer	z	Mean absolute value
16	Gyroscope	z	STD
17	Gyroscope	z	Median
18	Gyroscope	z	Waveform length
19	Accelerometer	z	Median
20	Gyroscope	x	Mean
21	Accelerometer	x	Mean absolute value
22	Accelerometer	x	Median
23	Accelerometer	y	Median
24	Gyroscope	z	Spectral peak index
25	Accelerometer	y	Mean absolute value
26	Gyroscope	x	Slope sign change
27	Gyroscope	y	Slope sign change
28	Accelerometer	x	Waveform length
29	Gyroscope	z	Mean absolute value
30	Gyroscope	x	Spectral peak index
31	Accelerometer	z	Mean
32	Accelerometer	y	Interquartile range
33	Accelerometer	y	Mean
34	Gyroscope	y	Mean
35	Accelerometer	x	Mean
36	Gyroscope	y	Median
